# Contextual organismality: Beyond pattern to process in the emergence of organisms

**DOI:** 10.1111/evo.13078

**Published:** 2016-10-27

**Authors:** Samuel L. Díaz‐Muñoz, Amy M. Boddy, Gautam Dantas, Christopher M. Waters, Judith L. Bronstein

**Affiliations:** ^1^ Center for Genomics and Systems Biology and Department of BiologyNew York UniversityNew YorkNew York10003; ^2^ Department of PsychologyArizona State UniversityTempeArizona85281; ^3^ Department of Pathology and Immunology, Center for Genome Sciences and Systems BiologyWashington University School of MedicineSt. LouisMissouri63110; ^4^ Department of Microbiology and Molecular Genetics, 5180 Biomedical Physical SciencesMichigan State UniversityEast LansingMichigan48824; ^5^ Department of Ecology and Evolutionary BiologyUniversity of ArizonaTucsonArizona85721

**Keywords:** Adaptation, conflict, cooperation, ecology, mutualism, organism, symbiosis

## Abstract

Biologists have taken the concept of organism largely for granted. However, advances in the study of chimerism, symbiosis, bacterial‐eukaryote associations, and microbial behavior have prompted a redefinition of organisms as biological entities exhibiting low conflict and high cooperation among their parts. This expanded view identifies organisms in evolutionary time. However, the ecological processes, mechanisms, and traits that drive the formation of organisms remain poorly understood. Recognizing that organismality can be context dependent, we advocate elucidating the ecological contexts under which entities do or do not act as organisms. Here we develop a “contextual organismality” framework and provide examples of entities, such as honey bee colonies, tumors, and bacterial swarms, that can act as organisms under specific life history, resource, or other ecological circumstances. We suggest that context dependence may be a stepping stone to the development of increased organismal unification, as the most integrated biological entities generally show little context dependence. Recognizing that organismality is contextual can identify common patterns and testable hypotheses across different entities. The contextual organismality framework can illuminate timeless as well as pressing issues in biology, including topics as disparate as cancer emergence, genomic conflict, evolution of symbiosis, and the role of the microbiota in impacting host phenotype.

Biologists have taken the definition of organism for granted for most of the history of the study of life. However, at a time when biologists have discovered that bacterial‐eukaryote symbiosis is near‐universal, that cancer cells may collaborate to create tumors, and that microbes can communicate and perform collective actions, the concept suddenly seems less clear. Did my genes stuck in my mother affect my brother's development (Boddy et al. 2015)? Are bacteriophages a part of our immune system (Barr et al. 2013)? Do cancer cells interact with normal cells to create tumors (Axelrod et al. 2006)? These empirical questions highlight the need to rethink and perhaps expand the definition of an organism, as well as to develop new conceptual frameworks that advance research on the evolution and persistence of organismality.

The organism has been traditionally defined using a checklist of properties, typically including response to stimuli, growth, and homeostasis (Huxley 1852; Wheeler 1911; reviewed in Santelices 1999; Pepper and Herron 2008). Wheeler (1911) provides an example of this type of traditional definition: “An organism is a complex, definitely coordinated and therefore individualized system of activities, which are primarily directed to obtaining and assimilating substances from an environment, to producing other similar systems, known as offspring, and to protecting the system itself and usually also its offspring from disturbances emanating from the environment.” (For a sampling of different definitions of the organism see Table 1 in Strassmann and Queller 2010.)

These traditional definitions served to identify and catalog the biological entities that were already understood to be organisms. The question of how organisms originate, however, had barely been addressed until the study of the major transitions in evolution (Buss 1987; Szathmáry and Maynard‐Smith 1995). This body of work suggested how organisms could emerge as an outcome of natural selection in evolutionary time: parts (e.g., cells) that once competed now function and evolve as a unit (e.g., a multicellular organism) characterized by high cooperation and low conflict (Szathmáry and Maynard‐Smith 1995). This hypothesis about the emergence of organisms inspired researchers to dispense with traditional definitions and to rely exclusively upon the criterion of cooperation and conflict as the basis for defining organisms (Queller and Strassmann 2009; Gardner and Grafen 2009; West et al. 2015). *Organismality* occurs when multiple biological entities interact to form a new entity characterized by adaptations, that is, an entity with “shared purpose” exhibiting high cooperation and low conflict among its parts (Queller and Strassmann 2009). Therefore, interactions among many cells that compose a human body meet the criterion of organismality, but so do certain groups composed of different individuals (ants forming a colony), different species (aphids and their bacterial symbionts), or different genes (viruses) (Queller and Strassmann 2009; West et al. 2015).

Queller and Strassmann's (2009) definition offers a novel way to identify new potential organisms; it is not limited to entities that we all can agree are organisms. This new definition generated a series of questions that stand as major challenges in the study of organismality (Strassmann and Queller 2010; West et al. 2015): What are the outcomes of interactions among the parts of a biological unit in ecological time? When do these interactions lead to organisms? What are the mechanistic details of these interactions? How and when does conflict appear within an already established organism? However, this more expansive definition does not provide the tools to answer these questions. A new conceptual framework is needed to address the challenges in the study of organismality.

The importance of addressing the challenges in the study of organismality goes beyond the philosophy of biology and increasingly is key to setting the research agenda in some of the most dynamic fields of biology today. Progress in studies of the eukaryotic microbiome (Youle et al. 2013), symbiosis (Moran 2007), organelle biology (Keeling et al. 2015), and cancer biology (Egeblad et al. 2010; Cleary et al. 2014; Aktipis et al. 2015), among others, requires making implicit or explicit decisions about what an organism is and selecting a framework to study all biological entities involved. Recent debate in the eukaryotic microbiome field highlights this issue: Should the collective host‐microbiota metagenome be the focus of study (Bordenstein and Theis 2015), or is the microbiota more commonly a distinct entity from the host (Moran and Sloan 2015)? Is the microbiota itself a collective entity or an assemblage of individual microbes in competition (Coyte et al. 2015)? The answers to these questions affect every level of the research down to the most fine‐grained details, such as the temporal resolution and depth of sampling strategies. An acknowledged organismality definition and framework can match the focus of the study with the question of interest, allowing many approaches to flourish. For example, in the case of cancer research, understanding cancer cells as a breakdown of multicellular cooperation (human organismality) can provide helpful insights into treatment. However, recognizing that some of these cancer subclones within the tumor may cooperate and produce public goods that benefit the entire tumor (tumor organismality) can lead to a contrasting evolutionary understanding of cancer (Cleary et al. 2014) and an entirely different approach to cancer therapeutics, such as blocking the shared public goods.

We suggest that the challenges facing the study of organisms can be met by using a new conceptual framework we call *contextual organismality*. We outline this framework, discuss its advantages and relationship to current frameworks, provide specific biological examples (Fig. 1), and discuss new insights that are gained from applying this framework that can help guide future research.

**Figure 1:**
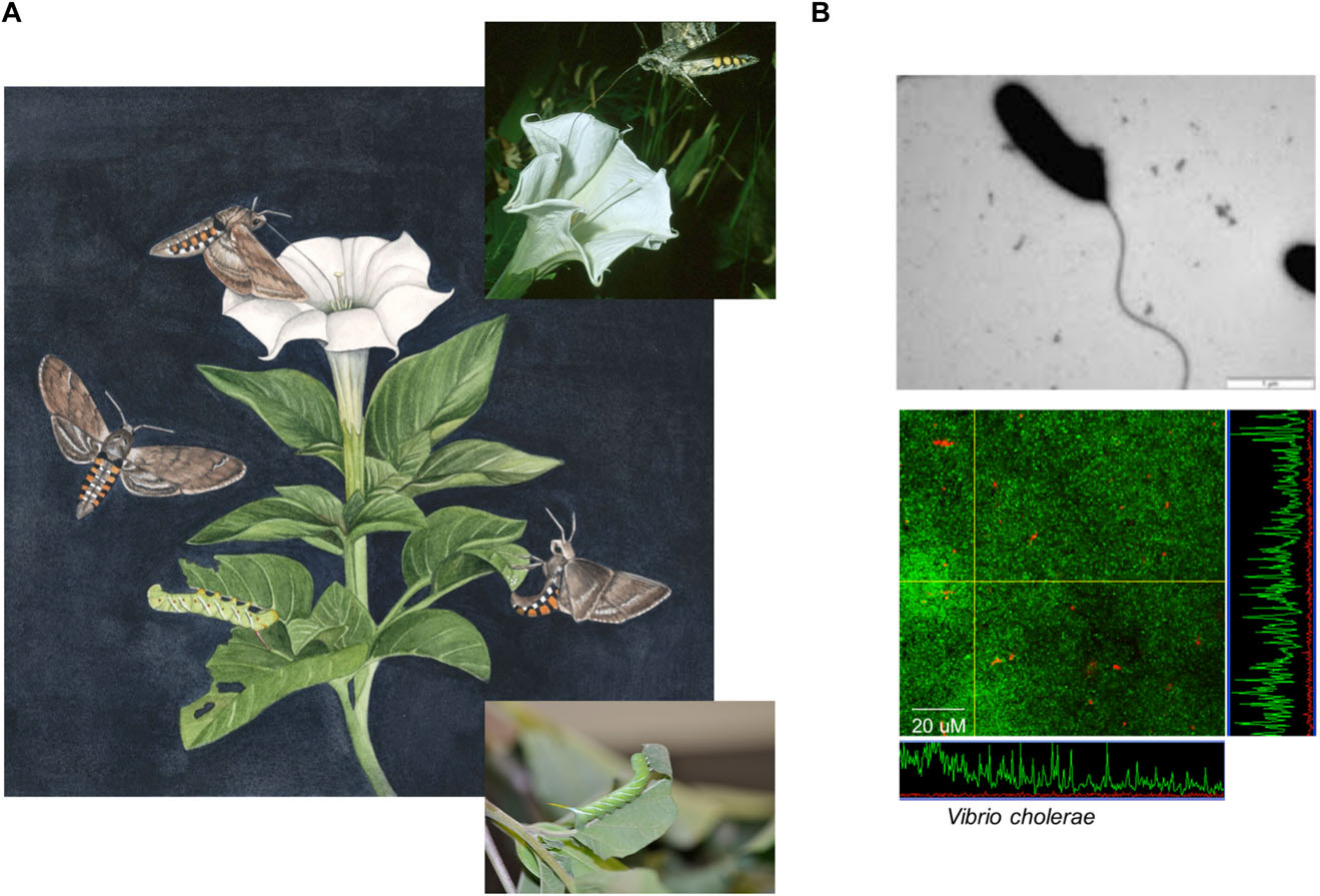
Examples of biological entities that show contextual organismality. Panel (A) shows an illustration of adult *M. sexta* hawkmoths collecting nectar and aiding pollination at *D. wrightii* plants and juvenile hawkmoths preying on *Datura* leaves. Insets show photographs of each stage. Panel (B) shows individual planktonic *Vibrio* bacterial cells moving independently (upper image) and a large number of *Vibrio* cells that communicate and coordinate secretions to create a biofilm (lower image). Image credits: Panel (A) main: Julie Johnson http://www.lifesciencestudios.com, Panel (A) insets: Robert Raguso and Judith L. Bronstein, Panel (B): Christopher M. Waters.

## The Framework: Organismality as a Dynamic Process

Contextual organismality begins from the recognition that the condition of organismality is not fixed but instead depends on context. Its goal is to elucidate the ecological contexts under which entities act as organisms. That is, it identifies when multiple biological entities (within or between species) form a new entity characterized by high cooperation and low conflict among the parts (Queller and Strassmann 2009) as a function of their current environment.

We argue that a given assemblage of parts will or will not behave as an organism‐like unit, depending upon the specific ecological conditions in time and space in which it occurs. Contextual organismality focuses on studying the traits and mechanisms that are associated with such transitions. For instance, single‐celled microbes in groups, widely regarded as competitors, can show highly cooperative interactions under conditions of starvation, leading to fruiting bodies that allow dispersal and survival of a subset of cells (Kaiser 2004). When and how these changes happen are the focus of the contextual organismality approach. Identifying *if* change happens is the starting point for the study of contextual organismality, as many units widely regarded as organisms will not in fact show context dependency. We discuss this useful distinction below (see *Reconciling Definitions: Organisms, Individuality, and the Major Transitions*).

The relevant contexts that determine a group's cooperation‐conflict dynamic vary according to its composition, but can include life stage (development), resource availability, population size, and interactions with other species. Examining different types of biological entities in different contexts reveals exciting new questions that emerge by viewing the phenomenon through the contextual organismality lens:

1. Does the group's cooperation‐conflict level change?
2. When and under what ecological context does it change?
3. What were the important traits that enabled that change?
4. Do different biological groups change under similar circumstances, or are similar traits important?

These questions form the basis of investigations guided by the conceptual organismality framework, as we will show in a subsequent section by applying them to biological examples (see *Contextual Organismality in Practice*). The answers to these questions can shed light on some of the major challenges in the study of organismality and also generate novel, testable hypotheses to gain further insights into interactions among biological entities.

Contextual organismality extends the study of organismality beyond current approaches, which employ binary definitions of the individual (West et al. 2015), identify the levels of selection (Michod 1997), or present a comparative view of the different examples of potential organisms (Queller and Strassmann 2009). Instead, the pragmatic approach of contextual organismality opens the door to the study of the *process of organismality* to understand organisms, paralleling the study of the *process of speciation* as part of understanding species.

## Contextual Organismality and Other Frameworks

To determine whether a particular entity exhibits organismality, groups are usually categorized by the level of cooperation and conflict, by meeting a threshold designating individuals (West et al. 2015), or by being assigned to a single point along a cooperation‐conflict graph (Fig. 1A; Queller and Strassmann 2009). Implicit in these categorizations is that the group is somewhat invariant or static. Alternatively, a group can be considered an organism when selection between groups is prevalent enough that selection within groups has been abolished (Gardner and Grafen 2009; Gardner 2015), or, in symbioses, when mutual dependency and strict vertical transmission exist (Estrela et al. 2016). The contextual organismality approach is agnostic toward the label (e.g., organism, individual), and instead focuses on the driving ecological processes. Thus, contextual organismality goes beyond a snapshot in evolutionary time to identify the ecological processes, mechanisms, and traits that solidify or dissolve organismality.

Some of the key elements of contextual organismality have been previously recognized in the literature. In discussing social insects, Ratnieks and Reeve (1992) warn against the use of “superorganism” applied categorically, instead arguing for “the pluralistic outlook, which would favor statements such as ‘foraging in the honey bee shows superorganismic properties’.” Strassmann and Queller (2010) hint at a similar distinction in the social insects: “***When*** conflict is strong enough, we would not consider the colonies to be organismal … ***When*** conflict is very low and cooperation very high, we think colonies should be viewed as organismal” [emphasis added]. Estrela et al. (2016) outline criteria to identify an organism (individual) within symbiotic interactions between species, but also point out that “Prior to such a transition, whether the interaction is parasitic, commensal, or mutualistic is a function of the balance between the net costs and benefits of association, which is contingent on the environment…” Our contextual organismality framework builds upon these proposals by developing a concept that can apply to all biological entities. Below, we explain how the contextual organismality framework can be applied in practice, provide examples, and highlight the new evolutionary and practical insights that arise from this perspective. As we outline below in the examples, researchers in different fields have in practice pursued many of the goals we outline for the contextual organismality framework.

## Contextual Organismality in Practice

**Figure 2:**
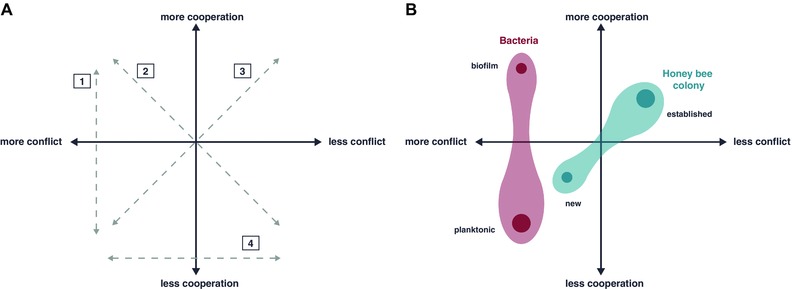
The cooperation‐conflict space is useful to visualize and evaluate potentially organismal interactions. Panel (A) illustrates organismality space (after Queller and Strassmann 2009) and some of the potential paths (numbered 1–4) organisms can move through under changing ecological contexts, such as development, resource availability, population size, and species interactions. In Panel (B), we provide examples of movement across organismal space in honey bee colonies (blue) and groups of microbial cells (red). In both examples, the cloud plot depicts the movement over “organismality space” and the labels represent the context that facilitates this change. The shading around the points is meant to convey the possibility of small changes in cooperation‐conflict in any context.

Context dependency is ubiquitous within mutualisms (cooperative interactions between species) in many forms (Chamberlain et al. 2014; Bruna and Hoeksema 2015). For example, the relationship between jimsonweed, *Datura wrightii* (Solanaceae), and the hawkmoth *Manduca sexta* shifts over developmental time with regard to the relative importance of cooperation versus conflict (Fig. 1A). As adults, the moths collect nectar at *Datura* plants, providing significant benefits (doubling seed and fruit production) due to pollen transport between flowers (Bronstein et al. 2009). As juveniles, however, the moths are voracious herbivores. Thus, the partners are mutualistic or antagonistic, simply based on the life stage of one of the two interacting species. Context changes the interaction, from low cooperation and high conflict in the moth's juvenile stage to high cooperation and low conflict in its adult stage (see Path 3 in Fig. 2A).

It is straightforward to envision that loose interactions, such as interspecific mutualisms, incorporate both cooperation and conflict and are context dependent. Indeed, context dependency is increasingly recognized to be a key feature driving the evolutionary dynamics of mutualism (Antonovics et al. 2015). However, do other more integrated units also show this characteristic? The following examples show how many groups exhibit context dependence and how this may indeed be a ubiquitous feature of organism‐like groups.

### HONEY BEE COLONIES

Honey bee colonies, long considered organisms (Wheeler 1911; Strassmann and Queller 2010) or superorganisms (Seeley 1989), also represent a case in which a shift in cooperation‐conflict levels occurs according to developmental timing (Fig. 2B). When a colony reproduces, potential honey bee queens are intensely aggressive and show no cooperation (Tarpy et al. 2004), as they engage in fatal physical combat to determine the reigning queen (Gilley 2001; Gilley and Tarpy 2005). After the sole queen is established, the group develops into a unit with low conflict and very high cooperation (Rangel et al. 2009). Thus, the ontogeny of a honey bee colony leads to a quintessential, cooperative superorganism, but it is born out of intense conflict. Existing research has answered most of the contextual organismality questions pertaining to honey bees: (1) The colony's cooperation‐conflict level changes (Tarpy et al. 2004); (2) these changes occur under specific biological and ecological circumstances (Robinson et al. 1989); and (3) are mediated by specific traits such as fighting ability and dispersal (Gilley and Tarpy 2005).

### SOCIAL AMOEBA

The social amoeba *Dictyostelium* shows a similar shift from conflict to cooperation among individuals of the same species, but within a different context: variation in resource availability. When resources are scarce and cells starve, swarming is activated to form a slug (Raper 1940). Cooperation ensues (Strassmann and Queller 2011) when some cells in the slug sacrifice their survival by becoming part of the base of a doomed multicellular stalk that propels cells dispersing from the fruiting body at the top of the stalk (Strassmann et al. 2000; Castillo et al. 2011). When resources are abundant, cells return to their unicellular state, where they hunt for bacteria independently (Raper 1940), experiencing high conflict and low cooperation with other *Dictyostelium* cells. A very complete example of the application of the contextual organismality framework is evident in recent work on *Dictyostelium*. First, researchers established that the relationship between the cells changes according to the environment and they studied the conditions that lead to the formation of the slug and the stalk (Ostrowski et al. 2008). Second, the traits that are important to facilitate or hinder this transition were established, down to the genetic level (Benabentos et al. 2009). Finally, studies have shown that slugs experiencing more conflict generate fitness costs to the slug (reduced mobility), and that those costs are differentially expressed in different environments (Foster et al. 2002; Castillo et al. 2005). Notably, these studies are not necessarily concerned with whether the stalk represents an organism, but instead focus on contextual changes in interactions among cells.

### BACTERIAL BIOFILMS

Variation in resource availability also induces a shift from cooperation to conflict in bacteria. Bacteria alternate between two general lifestyles, a motile planktonic state and a sessile community known as a biofilm. As individuals in well‐mixed populations, bacteria generally exist in a regime of high conflict with little cooperation, although there are certainly examples of cooperative behaviors (Bruger and Waters 2015). However, in a biofilm, spatial proximity to one's kin as well as sequestration of public goods leads to a higher degree of cooperation (Boyle et al. 2013). Biofilms also increase the height of bacterial communities to reach valuable nutrients when growing on a surface, essentially starving competitors, analogous to tall trees shading young saplings in a forest (Xavier and Foster 2007). This leads to a transition in the contextual organismality regime as illustrated in Figure 2B, and it is clear that bacteria can and do frequently move between these two quadrants.

The contextual organismality framework prescribes a focus on the traits enabling the biofilm‐planktonic transition and how they respond to environmental factors and adaptive fitness benefits to drive this transition. Quorum sensing, a collection of pathways mediating the detection and secretion of extracellular signals released by bacteria, is one key trait mediating the biofilm‐planktonic transition. Upon sensing of specific environmental cues, bacteria can use these signals to form a biofilm or to disperse from it, reentering the single‐cell state. Most bacteria, such as *Pseudomonas aeruginosa*, use quorum sensing to induce biofilm formation at high cell density (Davies et al. 1998). In contrast, quorum sensing in the genus *Vibrio* responds to the same environmental cue, high cell density, to represses biofilm formation (Hammer and Bassler 2003) due to a trade‐off in biofilms between local competition and dispersal (Nadell and Bassler 2011). Theory predicts these contrasting responses of biofilms to high cell density have distinct adaptive benefits, with some species *promoting* biofilm formation to outcompete other neighboring cells to access nutrients and other species *repressing* biofilm formation to facilitate dispersal and limit competition with their own lineage (Nadell et al. 2008). Placing these findings into a contextual organismality framework, the same environmental factor (high cell density) interpreted by a common trait (quorum sensing) can be associated with a transition to increased cooperation (biofilm formation) or decreased cooperation (biofilm dispersion), depending on the interactions among cells in the biofilm over time (Nadell et al. 2008). Viewing findings through this contextual organismality lens can elucidate general patterns across bacterial species, as well as uncover new modalities of biofilm formation.

Besides increasing our biological understanding of these processes, contextual organismality can also yield practical benefits. Because biofilm‐based infections are tolerant to antibiotic treatment and resist clearance by the host's immune system (Hall‐Stoodley and Stoodley 2009; Percival et al. 2010), there is much interest in interventions that disperse biofilm infections. Currently, each bacterial pathogen is viewed in a microcosm, and efforts are underway to identify the specific regulatory and mechanical factor that may drive dispersal for each species. However, the lens of contextual organismality can establish patterns across many bacterial species to identify fundamental signals (e.g., high cell density) promoting the switch from a cooperative organismal state to an individual free‐living state, leading to broad spectrum interventions that could target multiple pathogens (Boyle et al. 2013).

### CANCER TUMORS

The ontogeny of cancer tumors provides a fascinating example of complex organismality which changes over time. Building a multicellular body requires cooperation among somatic cells. Breakdown, or cheating on somatic cooperation, may result in cancer (Aktipis et al. 2015), leading to reduced cooperation at the level of the individual human. As a tumor develops within the host, multicellular cooperation amongst neoplastic cells may reemerge, leading to increased “organismality” in the developing tumor. Individual cancer subclones that interact cooperatively through cell–cell signaling have a selective growth advantage (Cleary et al. 2014). Additionally, only a subset of cancer cells is needed to provide signals for changes in the microenvironment that can then benefit the entire tumor, as observed in the signaling that stimulates a vascular network to supply resources to the tumor (Papetti and Herman 2002). Tumor tissue organization suggests additional components of cooperation, including within‐tumor cell communication to build patterns of organization similar to organ structures (reviewed in Egeblad et al. 2010). Thus, cancer initiation may be the result of intense conflict between neoplastic and somatic cells, but genetically diverse meta‐populations can cooperate within an advanced tumor (see review Tabassum and Polyak 2015). This process of contextual organismality in tumor cell formation could be described as traversing Pathway 3 in Figure 2A, followed by another shift along Pathway 1 to a region of high cooperation with high conflict. Understanding the contextual organismality process of tumor formation, for which types of cancers it occurs, and where individual cases fall on this spectrum, could guide therapeutic strategies that are most effective for targeting tumors at different stages of cooperation and conflict.

These examples illustrate that organismality is a context‐dependent feature of some groups, and show how consideration of the relevant contexts can illuminate the mechanisms at work in putative organisms. Contextual organismality provides a framework to unify these separate lines of research and highlight understudied questions. For instance, researchers who study social evolution in animals might not immediately think they study anything similar to cancer biologists and vice versa, but contextual organismality places their research in a common framework. It allows these two groups of researchers, employing disparate terminologies and pursuing different research priorities, to recognize commonalities in their work: they study how cooperation among biological entities changes into conflict over developmental time. This common language can encourage the adoption of new approaches across biological scales, as occurred when insights from social evolution theory were applied to cancer biology (Axelrod et al. 2006). The new social evolution perspective helped to explain the difficulties of traditional targeted treatment to tumors, namely that cooperation among different clones in a heterogenetic tumor can restore tumor growth (Cleary et al. 2014). In turn, this social perspective suggested novel avenues for treatment based on evolutionary principles, such as maintaining subclonal population diversity, including both therapy‐sensitive and therapy‐resistant subclones, to enhance competition for resources and slow the growth of the tumor, instead of eradicating it (Enriquez‐Navas et al. 2016). Similarly for other study systems, common patterns of movement between context‐dependent states varying in the degree of cooperation‐conflict can be related to the underlying processes that drive changes in organismality (Fig. 2A).

## Reconciling Definitions: Organisms, Individuality, and the Major Transitions

The contextual organismality framework is most readily applicable to facultative relationships, wherein changes in conflict‐cooperation levels are readily achieved under changing conditions in ecological time. In contrast, the organismality of entities that are unequivocally considered organisms, such as human individuals, is largely unaffected by ecological context. The “largely” qualifier is necessary, because particular circumstances such as pregnancy (Boddy et al. 2015) or autoimmune disease can increase conflict within a human, although not to the point of calling into question its existence as an organism (Strassmann and Queller 2010). That is, the cooperative and low‐conflict interactions among cells that make up human individuals persist under a wide variety of circumstances, unlike the cooperation of *Dictyostelium* cells to form a stalk, which only appears under specific conditions.

Thus, a lack of context dependency can be used as an indicator of an organism, that is, a group that preserves high cooperation and low conflict among the parts across widely divergent contexts. This conceptualization of the organism reconciles the fluidity of contextual organismality with organismality approaches that employ fixed definitions of an organism (Queller and Strassmann 2009), individual (West et al. 2015), or major transition (Buss 1987). An exciting question for future research is whether contextual organismality represents a stage that cooperative units pass through on the way to increased unification (see *Future Prospects*).

## What We Gain with Contextual Organismality

Beyond identifying organisms, the process of forming and maintaining an organism can be a subject of empirical inquiry. The recognition that organismality can be context dependent provides a road map for the ecological and mechanistic study of organismality. Contextual organismality is a complementary framework to current approaches to the study of organisms that offers distinct advantages—which we list below—to advance this field of study.

First, as we have outlined above, contextual organismality points to common patterns across disparate biological entities and, crucially, to the contextual features that influence the evolutionary process. Second, recognizing the existence of contextual organismality has the potential to direct us to what we do not know about interactions within and between organisms, to generate testable hypotheses. Finally, this approach has the advantage of being able to make—but not requiring—a binary decision on whether a particular interaction does or does not represent an organism (Queller and Strassmann 2009; West et al. 2015). The focus is on generating and testing hypotheses regarding the ecological circumstances that change interactions between biological entities. The appropriate ecological circumstances for each study system can be decided, but the framework can be applied across different fields, allowing field‐specific terminology and debates to be temporarily set aside to enable a greater understanding of the commonalities of interactions between biological entities. In this sense, contextual organismality provides a pragmatic framework that opens empirical inquiry into the process of organismality.

## Future Prospects

One of the challenges remaining to the study of organismality—including contextual organismality—is how we can quantify cooperation and conflict in a way that is comparable across systems, and accessible to the empiricist and the theoretician alike. Because the goal is to examine organismality across all life forms, a common metric or currency for measuring conflict and cooperation can prove elusive (Bronstein 2001). Fitness is the only unifying currency across all these systems. Theoretical frameworks (Gardner and Grafen 2009; Gardner 2015) and empirical measures (M. Roper, pers. comm.) are currently being developed to apply this concept in practice. For instance, Gardner and Grafen (2009) dispense with measuring cooperation and conflict, and instead measure whether selection between groups overwhelms selection within groups to determine if an entity constitutes an organism, a statement later framed as the “fundamental theorem of multilevel selection” (Gardner 2015). In this case (natural) selection is defined with respect to a “particular arena (biological population) and character (heritable portion of phenotype)” (Gardner 2015). That is, natural selection is contextual, in principle allowing comparisons to be made between groups of biological entities in different contexts. Thus, the contextual organismality framework can be applied to theoretical and quantitative approaches to meet challenges in the study of organismality.

The conceptual framework of contextual organismality also generates novel questions that represent potential avenues for future research. Below we discuss two of these questions, suggest potential study systems to address these novel questions, and indicate how research into these questions could change or support our current knowledge of organismality.

### IS CONTEXT‐DEPENDENT ORGANISMALITY A STEPPING STONE TOWARD INCREASED UNIFICATION?

Above we proposed that the lack of context dependency could be a new definition of the organism, with the corollary that context dependency was a stage that all different types of organisms potentially navigate. This is a testable hypothesis leading to the prediction that phylogenetically related biological entities with different degrees of unification will also differ in the context dependency of their interactions (in terms of cooperation‐conflict). Potential model systems to test this prediction are interspecific mutualisms that vary in their degree of unification (Estrela et al. 2016). Obligate symbionts and their hosts, by many definitions, meet the criteria to be considered organisms. Related symbionts that show decreased integration with the host should exhibit a more context‐dependent relationship with their hosts, in terms of the cooperation‐conflict dynamic. Alternatively, if there is within‐species variability in whether the host has symbionts and symbionts have the ability to live independently of hosts, host populations lacking symbionts (perhaps with access to the essential nutrient the symbiont provides) should show a context‐dependent relationship with a related symbiont. Our approach represents a departure from current studies of symbiosis, which attempt to demonstrate reciprocal selection to establish a coevolved symbiotic relationship; contextual organismality works “backward” to illuminate the ecological flexibility that precedes unified, coevolved, obligate symbioses (Estrela et al. 2016).

### DO DIFFERENT BIOLOGICAL GROUPS CHANGE UNDER SIMILAR CIRCUMSTANCES, OR ARE SIMILAR TRAITS IMPORTANT?

One of the goals of the contextual organismality approach is to enable comparisons between different biological entities by providing a common language and framework for phenomena now studied in isolation. As outlined in our examples, the cooperation‐conflict dynamic of a group can change in response to resource availability in a wide variety of biological entities. In response to nutrient starvation, bacteria, amoeba, and cancer cells increase their cooperative interactions to create new forms of organismality: fruiting bodies, slugs, and tumors, respectively. This insight of contextual organismality generates the testable hypothesis that nutrient limitation of cells—of any kind—in close proximity leads to collective action forming new multicellular structures that overcome starvation. A comparative approach in which studies would document responses of nutrient starvation of cells across the different kingdoms of life could be used to evaluate this hypothesis. If supported, this finding could potentially lead to unifying explanations (for instance, based on shared biophysical properties of cells) for the similar responses of cells to starvation across the tree of life.

It is our hope that the contextual organismality framework will contribute to expanding empirical and synthetic research into one of the fundamental questions of biology: “*What is an organism?*” As with many other fundamental questions, it is unlikely that a single answer will be forthcoming. However, tackling this question has great potential to generate knowledge. An analogy with the question “*What is a species?”* is appropriate (J. E. Strassmann, pers. comm.). After over three centuries of study, biology is arguably further away from an answer. Now there are multiple definitions, called species concepts. What has been gained is a breadth and depth in the knowledge of the *process* of speciation and all the different ways it can happen (De Queiroz 2007).

Likewise, it is our hope that contextual organismality serves as a useful framework for understanding the process by which organisms emerge. We advocate adding the important question “*When* is an organism?” to the research agenda. The answer lies in the mechanisms of organismality, which can inform issues as disparate as cancer emergence, the role of the microbiome, and genomic conflict, addressing both pressing and timeless questions in the study of life.

Associate Editor: R. Azevedo

Handling Editor: R. Shaw
